# Whole-genome microRNA sequencing analysis in patients with pulmonary hypertension

**DOI:** 10.3389/fgene.2023.1250629

**Published:** 2023-11-16

**Authors:** Shi Chen, Jinnan Zhong, Bingzhu Hu, Nan Shao, Chaosheng Deng

**Affiliations:** ^1^ Division of Respiratory and Critical Care Medicine, First Affiliated Hospital of Fujian Medical University, Fuzhou, Fujian, China; ^2^ Department of Respiratory and Critical Care, Wuhan No. 6 Hospital, Affiliated Hospital of Jianghan University, Wuhan, Hubei, China

**Keywords:** pulmonary hypertension, microRNA, transcriptome sequencing, differential expression, clinical diagnostic biomarkers

## Abstract

Pulmonary hypertension (PH) is a pathological disorder with multiple clinical manifestations that lead to cardiovascular and respiratory diseases in most patients. Recent studies have revealed that microRNAs (miRNAs) play important roles as upstream signaling molecules in several diseases, including PH. However, miRNAs that can be used as diagnostic or prognostic biomarkers for PH have not been identified. Thus, in this study, peripheral blood samples obtained from patients with PH and healthy individuals were subjected to genome-wide miRNA sequencing and transcriptome analysis. We screened 136 differentially expressed miRNAs in patients with PH and verified that four differentially expressed miRNAs, namely, hsa-miR-1304-3p, hsa-miR-490-3p, hsa-miR-11400, and hsa-miR-31-5p, could be used as clinical diagnostic biomarkers for pulmonary arterial hypertension. Our findings provide a basis for further in-depth investigations of the specific mechanisms of miRNAs in PH.

## 1 Introduction

Pulmonary hypertension (PH) is a pathological disorder with multiple clinical manifestations that lead to cardiovascular and respiratory diseases in most patients (Biener et al., 2023). It is characterized by a mean pulmonary arterial pressure of at least 25 mmHg (Lucke et al., 2019). PH can be primary or secondary to several other diseases. PH involves the constriction of blood vessels within the lungs, making it difficult for the heart to pump blood to the lungs (Barst et al., 2004). Over time, fibrosis develops in the affected blood vessels, which continues to increase pressure on the heart and affects blood flow. Additionally, increased heart workload can lead to right ventricle hypertrophy such that the heart cannot pump blood into the lungs, eventually causing right heart failure (Wideman et al., 2013). Vascular endothelial cells, smooth muscle cells, fibroblasts, and stromal cells are involved in PH development. Left-to-right shunt, which results in a continuous increase in pulmonary vascular flow and pressure, is one of the most common forms of PH. It leads to the abnormal proliferation of endothelial and smooth muscle cells, resulting in pulmonary vascular remodeling and eventually PH (Pan et al., 2011). Patients with PH often show symptoms such as fatigue, weakness, shortness of breath, abdominal distension, and, in severe cases, hepatomegaly and peripheral edema. If not actively treated, their prognosis remains poor (Lang and Marion, 2023).

Currently, the worldwide prevalence of PH is approximately 97 per million individuals, with a mortality rate of approximately 5–10 per 100,000 individuals. Hypoxia-induced PH is caused by lung diseases and other pathological factors that lead to hypoxia in the body (Wideman et al., 2013). Clinically prevalent in interstitial lung disease and chronic obstructive pulmonary disease, PH is often accompanied by deterioration of the patient’s exercise capacity, leading to a further increase in hypoxia and decrease in survival. Therefore, investigating the mechanisms underlying PH development is of significant clinical importance (Misaki et al., 2023).

The clinical treatment of PH is not highly effective. Among patients with PH, 75% are aged 20–40 years, whereas 15% are <20 years of age. The health of patients who do not receive timely treatment gradually worsens, and such patients have a short life expectancy (Humbert et al., 2014). Before the 1990s, the treatment options for PH were limited and patients had an extremely short survival time (Hoeper et al., 2002). However, with the continuous development of medical science, increase in treatment modalities, and development of new drugs, the 5- and 10-year survival rates of patients have considerably increased (Hackman and Lackner, 2006). Currently, the main treatments for PH involve drug therapy, surgery, and gene therapy. However, well-established drug therapy, surgery, and newly emerging gene therapy have some limitations (Walker et al., 2006). Some drugs such as diuretics, prostaglandin inhibitors, and phosphodiesterase inhibitors reduced mortality or even reversed PH in animal studies; however, they only improved PH symptoms without significantly decreasing the mortality (Pugh et al., 2013).

The histopathological changes in PH are characterized by pulmonary vasculature remodeling. These changes are generally accompanied by smooth muscle cell proliferation in the non-myelinated peripheral vasculature and common myelopathic arterial hypertension. Damage to small arteries and compensatory hyperplasia reduce the density of the arterial vasculature, leading to its comprehensive distension and proliferative changes, including thickening of the middle layer of the artery, cell proliferation, intimal fibrosis, and fibrinoid necrosis (Bouzina et al., 2021). Recent studies on PH pathogenesis have demonstrated that it is primarily related to the inhibition of voltage-dependent potassium channels in pulmonary artery smooth muscle cells; dysfunction of the pulmonary vascular endothelium, leading to imbalanced endothelial cytokine secretion; participation of inflammation in PH development and progression; dysregulation of elastase and matrix metalloproteinase secretion in PH, thereby promoting vascular remodeling by increasing extracellular matrix accumulation; and the molecular genetic mechanisms of PH (Simonneau et al., 2009).

MicroRNAs (miRNAs) are small, single-stranded RNAs comprising 18–25 nucleotides and are widely found in eukaryotes. miRNAs are highly conserved, tissue-specific, and temporally expressed; however, they lack an open reading frame and are mostly noncoding. They suppress protein synthesis by inhibiting the translation of specific mRNAs or directly degrading mRNAs (Eulalio et al., 2008). As miRNAs function in almost all physiological and pathological processes, they can exert their effects in various physiological and pathological states (Vickers et al., 2013).

Recent studies have revealed that miRNAs play important roles as upstream signaling molecules in PH. Combination of multiple drugs to reverse PH symptoms has become a popular research topic (Khandagale et al., 2022). Detecting and regulating the expression of PH-associated miRNAs may help prevent or treat PH. Therefore, miRNAs are important starting points to identify novel therapeutic targets for PH (Liu et al., 2021).

To this end, in the present study, the peripheral blood samples of patients with PH were subjected to genome-wide miRNA sequencing and transcriptome analysis. By comprehensively analyzing the differentially expressed miRNAs in PH, we assessed the correlation between miRNAs and PH development, predicted their target gene loci, and elucidated the correlation between miRNAs and PH. Our findings provide a new reference and basis for future in-depth investigation of the specific miRNA-related mechanisms in PH.

## 2 Materials and methods

### 2.1 Sample collection and RNA extraction

Peripheral blood was collected in 10-mL heparin anticoagulation tubes from eight volunteers at Wuhan Sixth Hospital. All volunteers provided written informed consent. Sterile phosphate buffer (pH 7.4) was diluted in an equal volume and centrifuged at 3000 × *g* for 5 min. A pipette was used to aspirate the supernatant, which was rapidly frozen in liquid nitrogen for 1 h and then incubated at −80°C or stored in liquid nitrogen for long-term storage.

For RNA extraction, TRIzol was added to the blood samples at a 1:3 ratio and thoroughly mixed. Thereafter, the sample was thoroughly blown with a pipette and shaken until complete dissolution of the floc, followed by incubation at room temperature for 5 min to completely degrade the nucleoprotein body.

### 2.2 RNA detection, library construction, and sequencing

The RNA samples were quantified using various methods. Nanodrop was used for determining RNA purity (OD_260_/_280_ ratio) and Qubit for precisely quantifying RNA concentration. The Agilent 2100 bioanalyzer was used for precisely detecting RNA integrity and indicators such as RNA integrity number, 28S/18S, presence or absence of uplift at the baseline of the graph, and 5S peak. RNA electrophoresis was used to detect whether the sample was diffuse or contaminated with genomic DNA.

After passing the sample testing, the Small RNA Sample Pre Kit was used to construct libraries using the special structures of the 3′ and 5′ ends of the small RNA (sRNA), i.e., the complete phosphate group at the 5′ end and hydroxyl group at the 3′ end. Using total RNA as the starting sample, sRNAs were directly synthesized by adding connectors to both ends. Thereafter, reverse transcription was performed for cDNA synthesis. After polymerase chain reaction (PCR) amplification, the target DNA fragments were separated via polyacrylamide gel electrophoresis. The cDNA libraries were obtained by cutting and recovering the amplicons from the gel. The quality of the constructed libraries was tested, with all requirements met before the library was sequenced. The following instruments were used for quality testing: 1) Qubit 2.0 fluorometer for accurate quantification and 2) the Agilent 2100 bioanalyzer for library size detection. After the library size met the required expectations and the libraries passed the tests, they were sequenced using the Illumina platform based on the effective concentration and target downstream data volume.

### 2.3 High-throughput miRNA sequencing

Eight miRNA transcriptome sequencing libraries were constructed by the Frasergen Company for the healthy and PH groups. The quality of the raw sequencing data was evaluated, and the raw data (raw reads) were filtered using Seqtk software to obtain high-quality data (clean reads). These clean reads were compared with the reads in miRBase, a human genome database used to identify known miRNAs. The unknown sRNAs were compared with the RNAs in Rfam database, a database comprising noncoding RNAs (ncRNAs). Thereafter, the sRNA fragments were annotated and counted, and the unknown small rRNAs were predicted using miRDeep software based on the signature hairpin structures of the miRNA precursors.

The following analysis was performed by taking the average of the two sample groups, which were divided into the CK (healthy) and PH groups for comparison. The reads from each sample were compared using miRBase to determine miRNA expression (in counts per million [CPM]). The expression was screened using CPM to calculate the metrics (CPM). The DESeq software was used to perform differential expression analysis between the PH and CK groups. miRNAs with a *p*-value of ≤0.05 and log2 (foldchange [FC]) of ≥1 were selected as the differentially expressed miRNAs. The transcriptome data has been submitted to the National Genomics Data Center (https://ngdc.cncb.ac.cn/; GSA No.: HRA007288).

### 2.4 Volcano map and clustering analysis of the differential miRNAs

The differentially expressed miRNAs were analyzed. First, to visualize the distribution of the false discovery rate and differential FC of the differential miRNAs between the two sample groups, a volcano plot comparing the two sample groups was constructed. Second, to visualize the expression patterns of the differential miRNAs in all samples, expression pattern and clustering analyses were performed.

### 2.5 Prediction of the target genes of the differentially expressed miRNAs and gene ontology (GO) and kyoto encyclopedia of genes and genomes (KEGG) enrichment analysis

The online prediction software DAVID bioinformatics Resources 6.7 (https://david.ncifcrf.gov/) was used to functionally predict the target genes of the miRNAs, including GO terms and KEGG pathway enrichment analysis. The predicted target genes were enriched using GO and KEGG to analyze the functions and signaling pathways of the target genes and to elucidate the potential functions of the differentially expressed miRNAs (Ashburner et al., 2000; Kanehisa et al., 2004).

### 2.6 Quantitative reverse transcription-polymerase chain reaction (qRT-PCR) validation

To verify the accuracy of transcriptome sequencing, four differentially expressed miRNAs in the PH group compared with the CK group were detected using qRT-PCR to verify the relative expression. 1) The upstream primers for qRT-PCR were designed based on the mature sequences of the four miRNAs, whereas the downstream primers were provided by the miRcute Plus miRNA qPCR Kit (Tiangen FP411). The qRT-PCR primer sequences were designed according to the instructions of the reagent kit and synthesized by Shanghai Bioengineering Biotechnology Service Co. 2) cDNA was prepared using 2× miRNA RT reaction, 10 μL buffer, 2 μL of miRNA RT Mix, 500 ng of RNA, and RNase-free water in sterilized Eppendorf tubes to a final volume of 20 μL. The product was reverse transcribed at 42°C for 60 min and 95°C for 3 min and then stored at −20°C. 3) For real-time quantitative analysis of the relative expression of the four miRNAs using the SYBR Green dye method, cDNA was used as the template and U6 was used as the internal reference gene. The total reaction volume for qRT-PCR was 10 μL and comprised the following components: 5 μL of 2× mix, 1 µL of the amplification primer 1 (2 μM), 1 µL of the reverse primer (2 μM), 1 μL of cDNA, and 2 µL of ddH_2_O. The mixture was amplified under the following conditions: 50°C for 2 min; 95°C for 10 min; 40 cycles at 95°C for 15 s and 60°C for 1 min; and then 95°C for 15 s, 60°C for 15 s, and 95°C for 15 s. Three replicates were used for each sample. The relative expression of the target genes was calculated using the 2^−ΔΔCT^ method, where ΔCt = Ct target gene − Ct internal reference gene, CK-1 was used as the control sample, and ΔΔCt = ΔCt experimental sample −ΔCt control sample. The sequencing results were compared using Excel.

## 3 Results

### 3.1 Comparison of sRNA sequencing data for patients with PH and healthy controls

**FIGURE 1:**
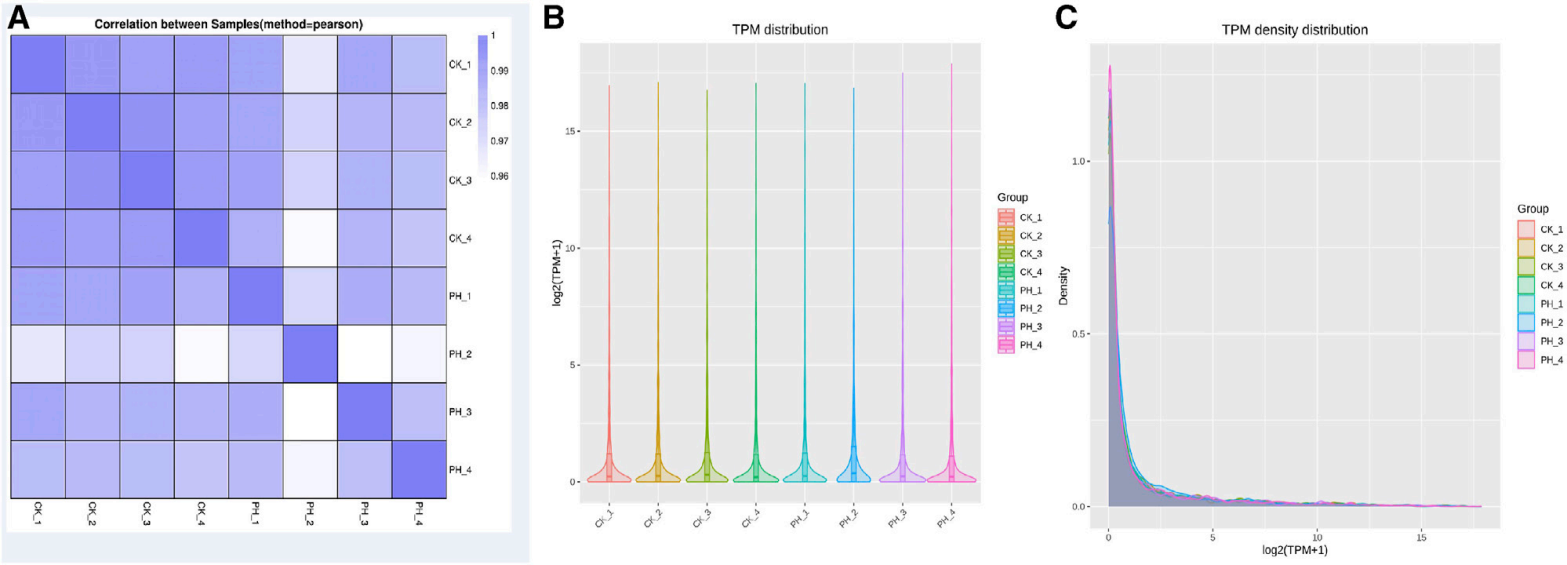
Comparison of small RNA sequencing data for 4 CK samples and 4 PH patient samples. **(A)** Correlation analysis of 8 samples; **(B)** miRNA TPM distribution analysis of 8 samples; **(C)** miRNA TPM density distribution analysis of 8 samples.

The correlation of miRNA expression between samples is an important index to assess the reliability of an experiment and whether the sample selection is reasonable. The closer the correlation coefficient is to 1, the higher the similarity of expression patterns between samples. The correlation coefficients of intra- and inter-group samples are calculated based on the expression of all genes in each sample, and a heat map is drawn, which can directly demonstrate the sample differences between groups and the repetition of the samples within groups. The correlation between the miRNA expression in healthy controls and patients with PH is shown in Figure 1A. The correlation between the samples in the groups was good; however, the correlation between the groups was lower than that between groups, indicating that the data can used for differential miRNA analysis.

Determining TPM is a method for analyzing the expression of miRNA. It can determine the amount of gene expression by calculating the TPM value of each miRNA. TPM value refers to the percentage of each miRNA in the total RNA. It can reflect gene expression and is its commonly used unit. The advantage of the TPM method is that it can eliminate the differences between samples to compare the gene expression of different samples. Additionally, TPM can be used to compare the expression of different genes to determine which genes have the greatest expression in different samples. However, the miRNA TPM and TPM density distribution analysis of the eight samples revealed that the overall differences were not significant (Figures 1B, C).

### 3.2 Whole-genome miRNA sequencing analysis

**FIGURE 2:**
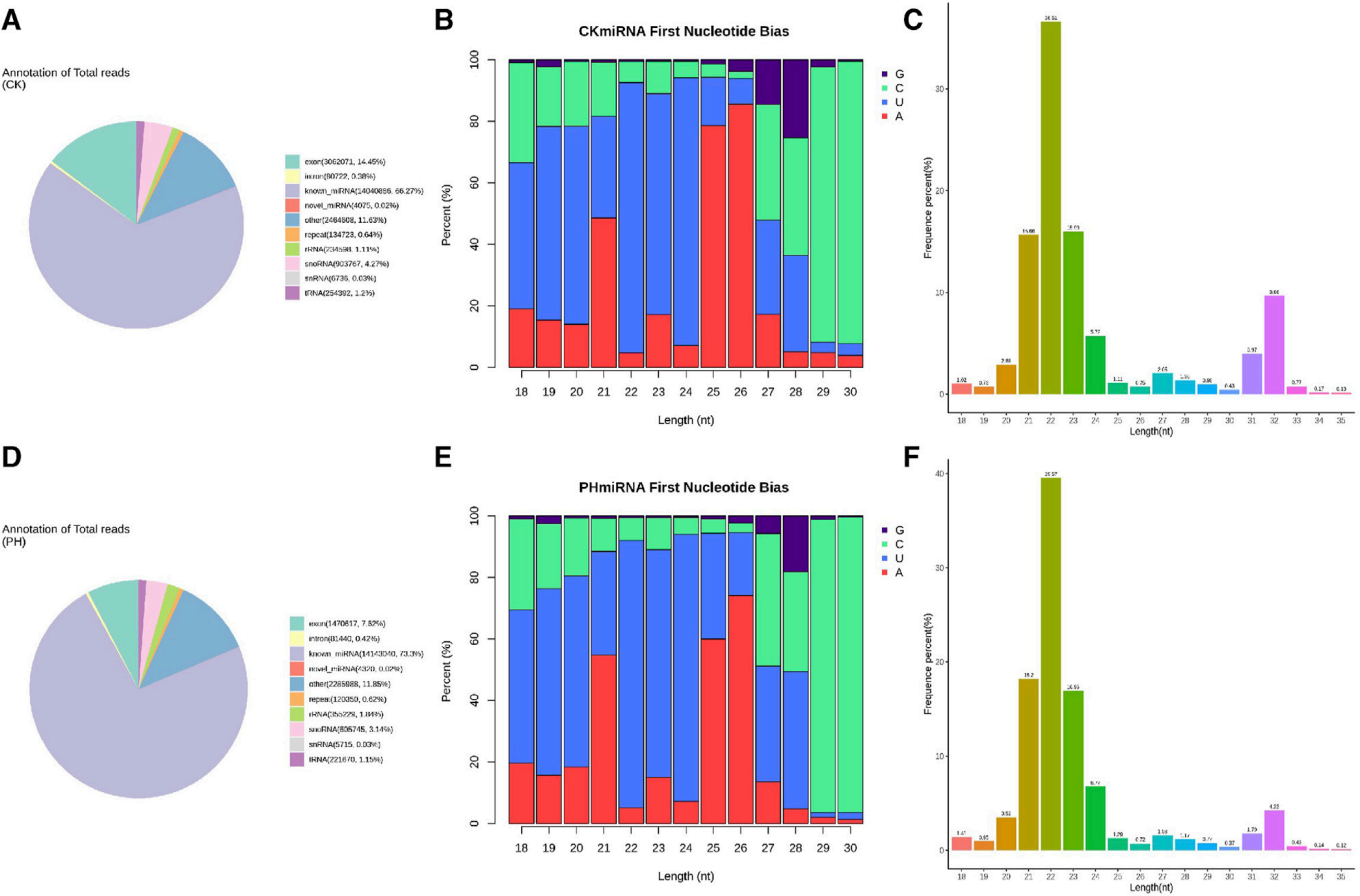
Sequence analysis of whole genome miRNA sequencing. **(A)** Small RNA classification annotation statistics and genomic site analysis of the CK group; **(B)** The small RNA first base and base distribution at various positions of mature miRNAs of the CK group; **(C)** The small RNA length distribution statistics of the CK group; **(D)** Small RNA classification annotation statistics and genomic site analysis of the PH group; **(E)** The small RNA first base and base distribution at various positions of mature miRNAs of the PH group; **(F)** The small RNA length distribution statistics of the PH group.

We compared the clean reads of mature miRNAs, miRNA hairpins, mRNAs, mature and primary tRNAs, small nucleolar RNA (snoRNAs), rRNAs, other ncRNAs, and (optional) known RNAs using bowtie (Langmead and Salzberg, 2012). The ncRNAs were removed as much as possible to enable miRNA detection and prediction. The results showed that 66.27% of the sRNAs sequenced in the CK group were mapped to known miRNA sites in the genome, 14.45% to exons, and 4.27% to the snoRNA loci (Figure 2A). Of the sRNAs sequenced in the PH group, 73.3% were mapped to the known miRNA loci in the genome, 7.62% to exons, and 3.14% to the snoRNA loci (Figure 2D).

miRNA precursors are cleaved by Dicer to form mature miRNAs, and the restriction sites mark the first base of the mature miRNA sequence. Therefore, the distribution of the first base of the sRNAs and position of the mature miRNAs were compared and analyzed (Figures 2B, E). The results showed that the miRNAs in the CK and PH groups had highly similar first base preference.

For the clean reads of each sample, sRNAs of a certain length were selected for further analysis. The results showed that the sRNAs in the CK and PH groups had a highly similar length distribution (Figures 2C, F).

### 3.3 Expression pattern cluster analysis of the differentially expressed miRNAs

Generally, genes encoding miRNAs whose expression differs by > 2-fold in different groups are differentially expressed. Thus, setting the criteria for screening differential genes is critical. We used |log2 (FC)| >1 and Padj <0.05 as the criteria for identifying differentially expressed genes.

**FIGURE 3:**
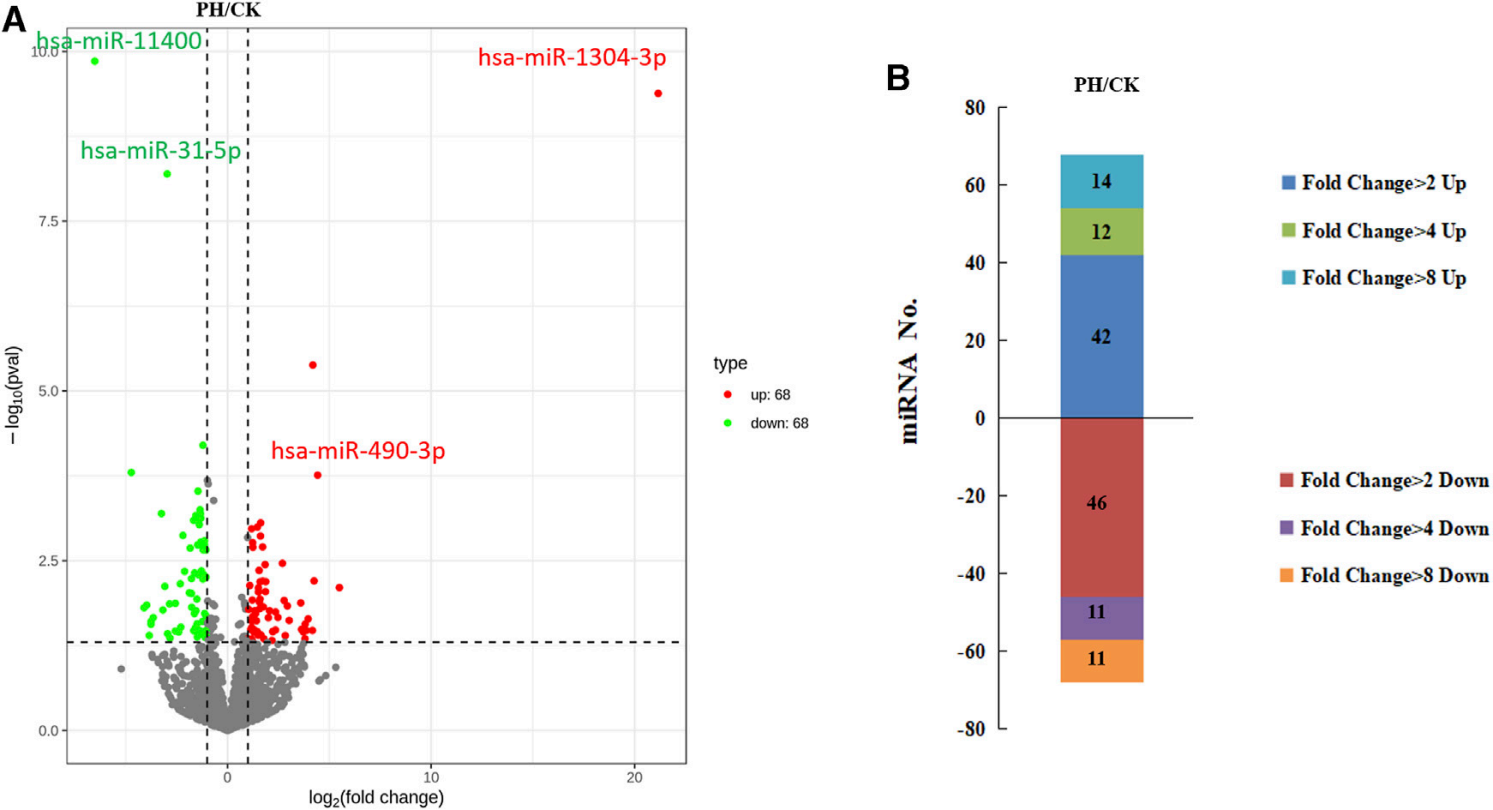
The differential expression miRNAs. **(A)** Volcano map of differentially expressed miRNAs; **(B)** The fold change of differentially expressed miRNAs.

**FIGURE 4:**
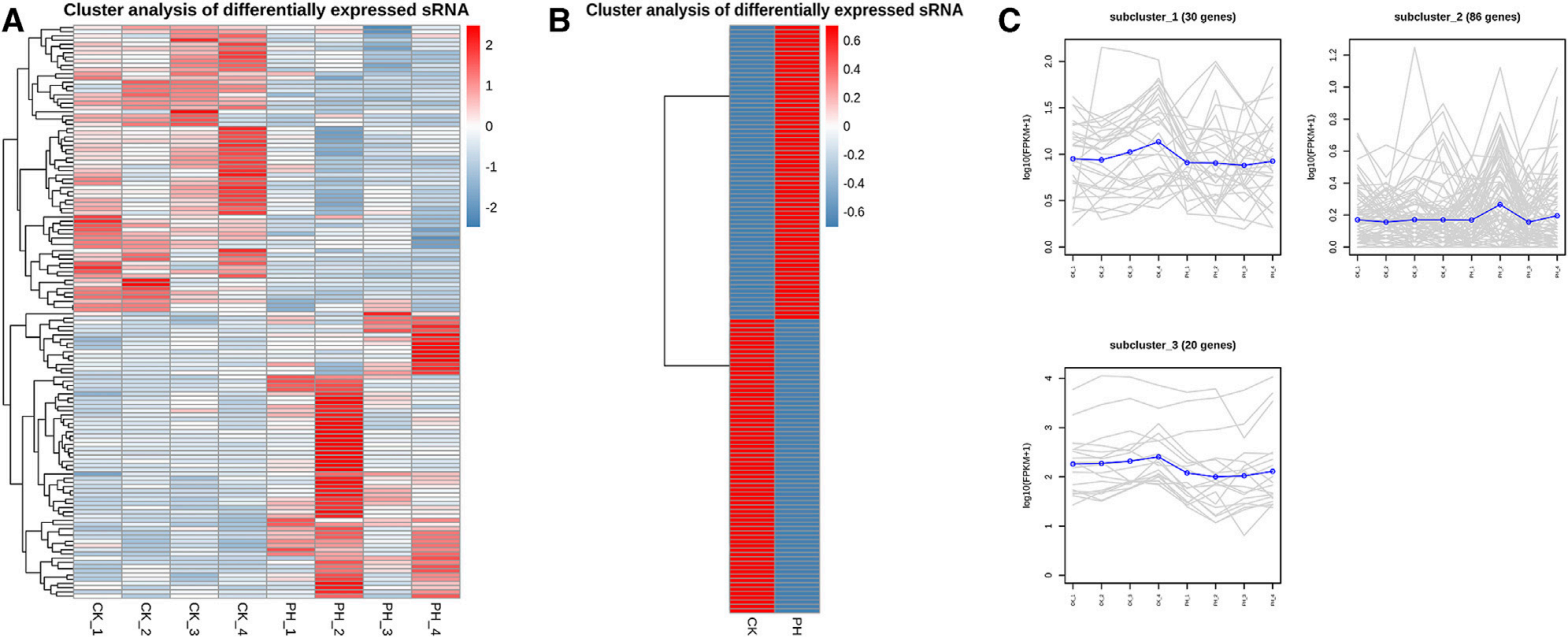
Expression patterns cluster analysis of differentially expressed miRNAs. **(A)** Expression cluster analysis of differentially expressed miRNAs in 8 samples; **(B)** Expression cluster analysis of differentially expressed miRNAs in CK group and PH group; **(C)** Expression patterns cluster analysis of differentially expressed miRNAs in 8 samples.

The volcanic map revealed 136 miRNAs with >2-fold differential expression in the PH group, of which 68 were upregulated and 68 were downregulated (Figure 3A). The numbers of upregulated and downregulated miRNAs showing differences between 2- and 4-fold, 4 and 8-fold, and >8-fold in the PH group were 42 and 46, 12 and 11, and 14 and 11, respectively (Figure 3B). A heat map (Figure 4A) and a line graph (Figure 4C) show the pattern of the differentially expressed miRNAs and their clustering in eight samples. Additionally, the difference in miRNA expression between the CK and PH groups was clustered using a heat map (Figure 4B).

### 3.4 GO functional and KEGG pathway enrichment analyses of the genes targeted by the differentially expressed miRNAs

**FIGURE 5:**
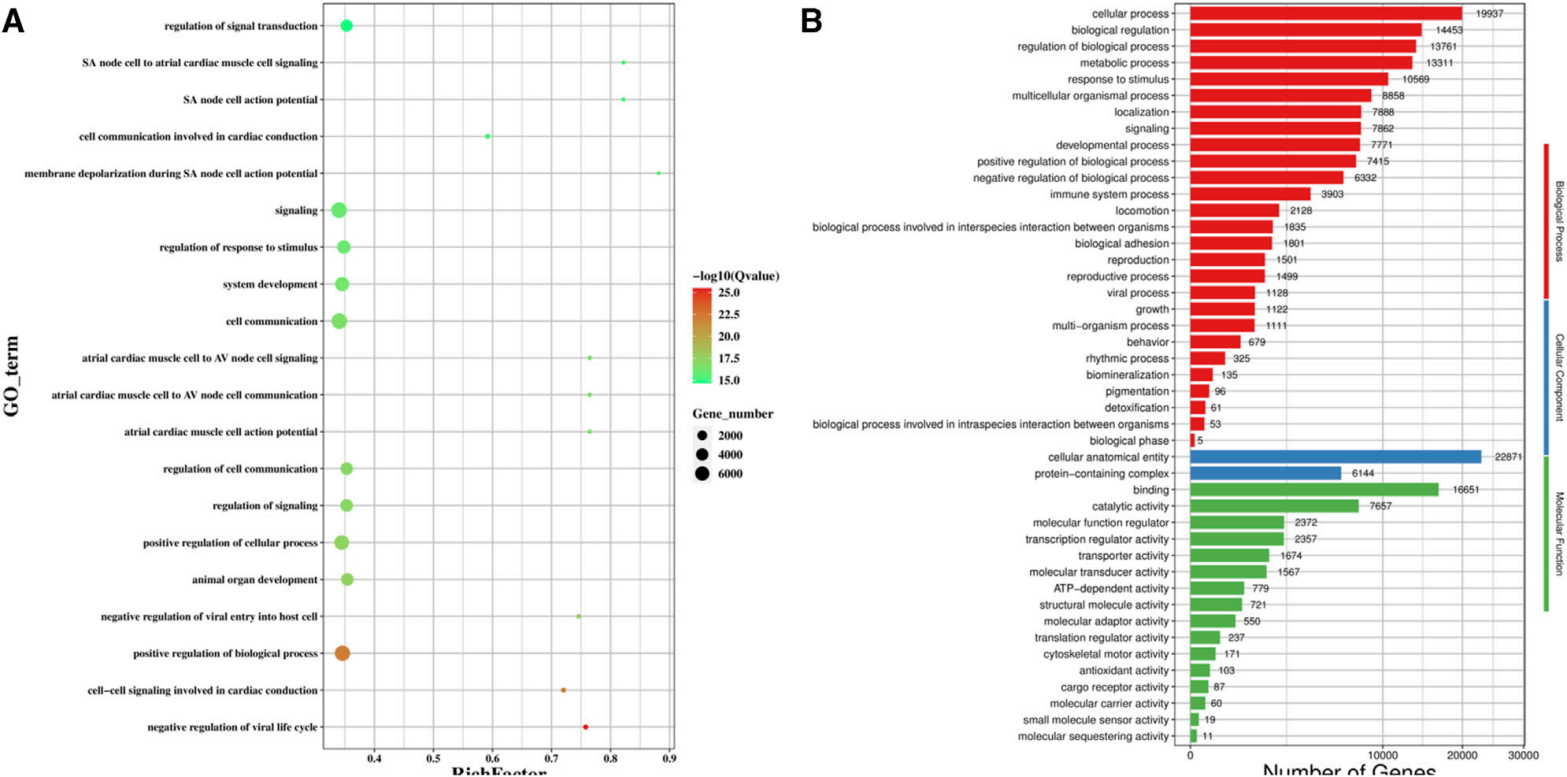
GO functional enrichment analysis of the target gene of differentially expression miRNA. **(A)** Scatter plot of GO enrichment analysis of the differentially expression miRNA target gene; **(B)** GO enrichment histogram analysis of the differentially expression miRNA target gene.

We predicted the genes targeted by the differentially expressed miRNAs using MiRanda and qTar, and the genes at the intersection of the two software were shortlisted for GO classification and enrichment analysis (Figure 5). GO analysis (Figure 5A) revealed that the GO terms for the top five differentially expressed genes were signaling, cell communication, positive regulation of biological process, positive regulation of cellular process, and regulation of response to stimulus. Among the three categories of GO enrichment analysis, the top three GO terms for the biological process were cellular process, biological regulation, and regulation of biological process; the two GO terms enriched for the cellular component were cellular anatomical entity and protein-containing complex; and the top three GO terms for molecular function were binding, catalytic anatomical entity, and molecular function regulator (Figure 5B).

**FIGURE 6:**
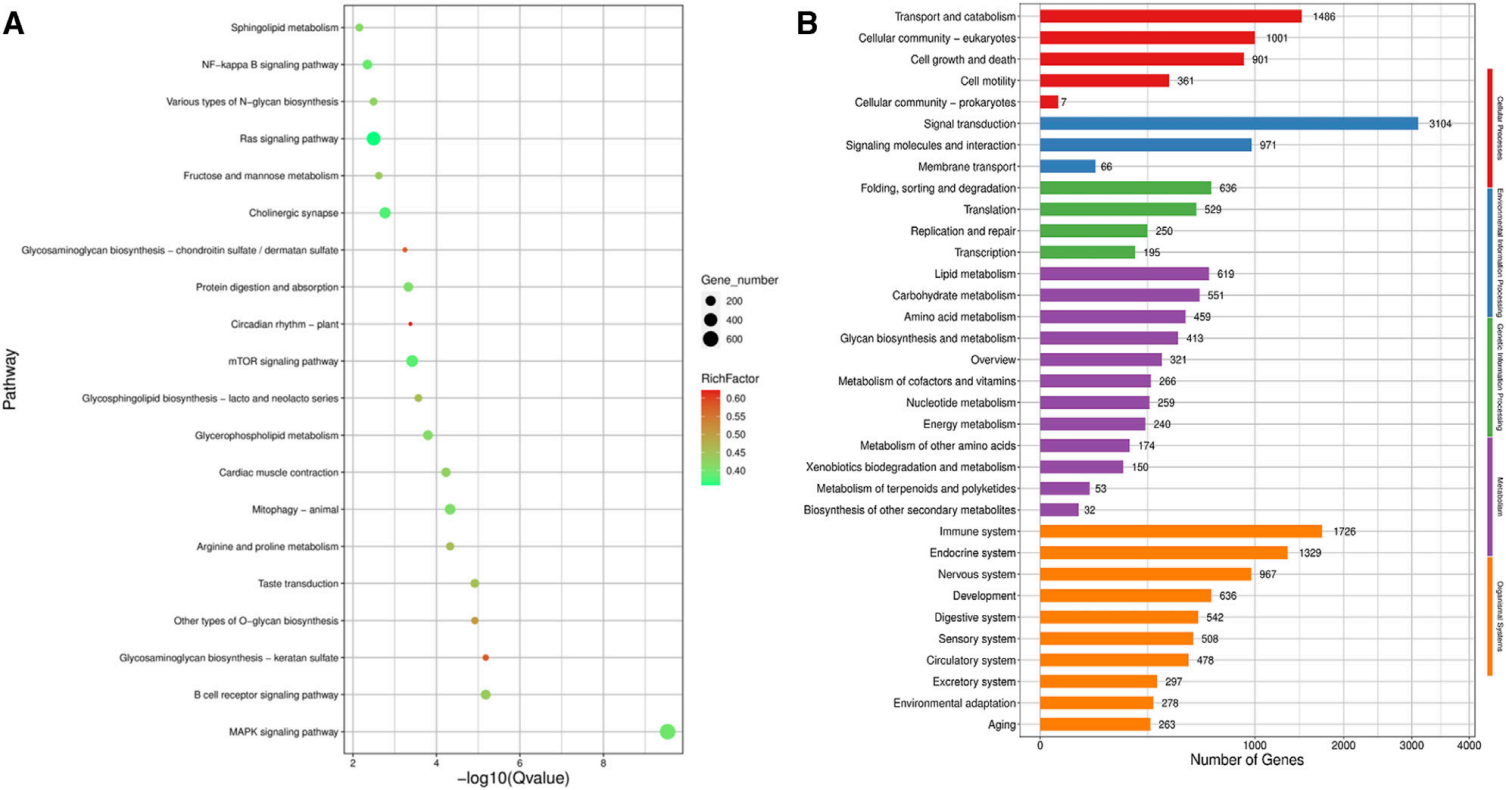
KEGG pathway enrichment analysis of the target gene of differentially expression miRNA. **(A)** Scatter plot of KEGG pathway enrichment analysis of the differentially expression miRNA target gene; **(B)** KEGG pathway enrichment histogram analysis of the differentially expression miRNA target gene.

KEGG pathway analysis (Figure 6A) revealed that the top five pathways related to the differentially expressed genes were MAPK signaling, Ras signaling, mTOR signaling, cholinergic synapse, and NF-kappa B signaling pathways. Among the five categories of KEGG pathway enrichment analysis, the top three terms for the differentially expressed genes related to cellular processes were transport, catabolism, and cellular community-eukaryotes; the top three terms related to environmental information processing were signal transduction, signaling molecules and interaction, and membrane transduction; the top three terms related to genetic information processing were folding, sorting, and degradation; translation; and replication and repair; the top three terms related to metabolism were lipid, carbohydrate, and amino acid metabolism; and the top three terms related to organismal systems were immune, endocrine, and nervous systems (Figure 6B).

### 3.5 Validation of the differentially expressed miRNAs and their potential as diagnostic biomarkers for PH

We selected two upregulated miRNAs (hsa-miR-1304-3p and hsa-miR-490-3p) and two downregulated miRNAs (hsa-miR-11400 and hsa-miR-31-5p) (Figure 3A) in the PH group based on their abundant and differential expression multiple (recognizable). We performed qRT-PCR for these four miRNAs to validate our transcriptome data and determine the feasibility of using them as clinical diagnostic markers for PH.

**FIGURE 7:**
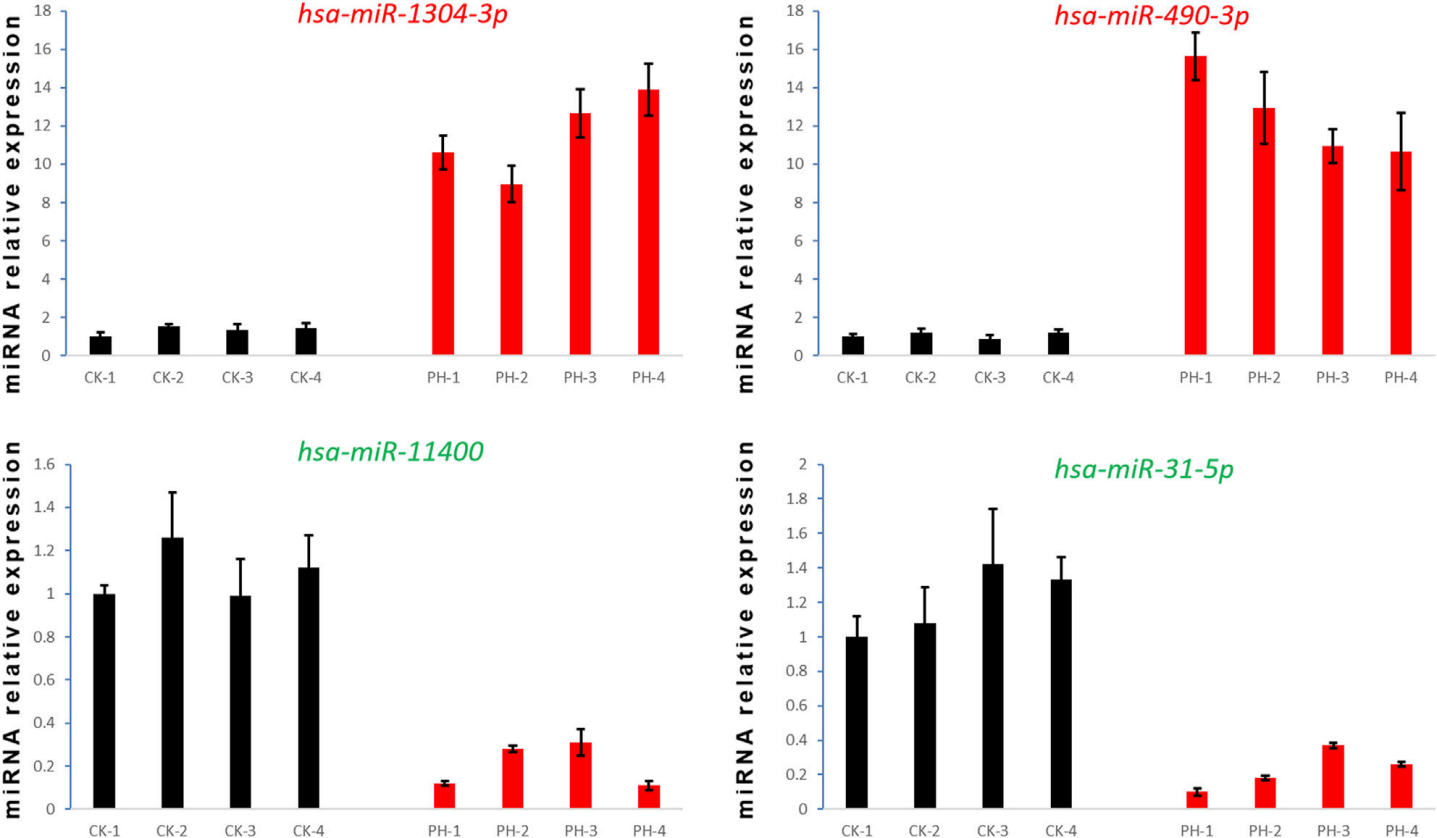
Detection of four miRNA expression levels by qRT- PCR.

qRT-PCR confirmed that the expression of hsa-miR-1304-3p and hsa-miR-490-3p was significantly upregulated, whereas that of hsa-miR-11400 and hsa-miR-31-5p was significantly downregulated, in the peripheral blood of all patients with PH compared with the CK group (Figure 7). Collectively, these findings prove the reliability and validity of our data and provide evidence that these four differentially expressed miRNAs are potential diagnostic markers for PH.

## 4 Discussion

PH is a serious and life-threatening disease of the cardiovascular system caused by an abnormal increase in pulmonary vascular resistance, persistent hyperplasia of the intima and media of the pulmonary arteries, and eventual vascular remodeling that leads to thrombosis *in situ*, right ventricular hypertrophy, and right heart failure, which may lead to death (Aldashev et al., 2000). Studies have demonstrated that the average survival of patients with PH is only 1–9 years after the onset of symptoms and even shorter in patients with right heart failure. Furthermore, effective treatments are lacking, resulting in poor patient prognosis (Liu et al., 2006). Although some progress has been made in PH prevention and treatment in recent years, the molecular mechanism underlying PH remains unclear. Furthermore, current treatment methods fail to conclusively improve the pathological state of PH. Thus, it is important to determine the molecular mechanisms underlying PH pathology for devising more effective treatment modalities (Sommer et al., 2021).

MiRNAs are small single-stranded nucleotide RNAs approximately 22 nt in length. They are present in a variety of organisms and play an important role in the regulation of gene expression. miRNAs are key regulatory RNAs in many respiratory diseases and can participate in the pathophysiological processes of respiratory diseases by regulating multiple signaling pathways. Asthma is a respiratory disease in which miRNA function is relatively well studied; for example, miR-192-5p and miR-155 overexpression is involved in asthma development and promoting allergic cell activation (Chen et al., 2017; Lou et al., 2020). Two recent independent studies proposed and validated that miRNA-26a-5p may influence asthma pathogenesis by regulating downstream target genes through the identification of differential miRNAs in humans and mice, suggesting that the functional identification of differential miRNAs by reverse genetics is also an effective method for determining the correlation between diseases and related miRNAs (Roffel et al., 2022; Zhong et al., 2022). Therefore, an in-depth study of miRNAs associated with pulmonary hypertension can help explore the pathogenesis of this condition and find new ways for its early diagnosis and treatment.

Owing to its vascular pleiotropic nature, PH may act as an upstream regulatory molecule in the PH signaling pathway. Several adverse factors associated with PH, including hypoxia and inflammation, regulate miRNA expression in vascular cells. So far, very few miRNAs have been reported to be involved in PH onset and development.

Earlier, miRNA51, miR-322, and miR-322 were co-expressed in animal models of hypoxia- and wildtype lilybin-induced PH. However, these miRNAs were not comparably expressed in the lung tissues of rats with PH (Caruso et al., 2010). Because of the differences between these two PH models, they also showed some unique differentially expressed miRNAs. For example, let-7a and miR-21 were expressed at significantly lower levels in the lung tissue of rats with wild lily lye-induced PH; thus, they may play key roles specific to the development of wild lily alkaloid-induced PH (Izumiya et al., 2015). PH onset is a chronic and dynamic process, with temporal pathological changes being associated with specific miRNAs. Studies have suggested that miR-204, which is dysregulated in several PH models, may affect pulmonary vascular remodeling by regulating the downstream target gene Src-homology 2 domain-containing phosphatase 2. In a rat model of hypoxia- and wild lily lye-induced PH, inhibiting miR-17 expression increased the expression of cell cycle protein-dependent kinase inhibitor 1A, which in turn reduced right ventricular systolic pressure and improved pulmonary vascular remodeling (Margaillan et al., 2013). Through GO and KEGG pathway analyses of the genes targeted by the differentially expressed miRNAs, signal transduction and immune-related pathways were found to be involved in PH.

The expression of many miRNAs is strongly correlated with disease severity. Thus, miRNAs can be used as diagnostic markers for early disease detection. Because miRNAs are stable in blood, it is easy to identify their expression profile in serum or plasma samples in diseased conditions. At present, miRNA biomarkers have been proposed for many diseases, including miR-423-5P for distinguishing lesion status in heart failure (Bauters et al., 2013), miR-126 for endothelial diabetes mellitus, and miR-126 for type II diabetes mellitus. In endothelial diabetes, miR-126 expression is decreased. However, the isolation and precise quantification of miRNAs in the peripheral blood remains challenging (Liu et al., 2014). Additionally, correlating miRNA expression with various pathological stages requires further investigations. Our results showed that upregulated levels of hsa-miR-1304-3p and hsa-miR-490-3p and downregulated levels of hsa-miR-11400 and hsa-miR-31-5p may have clinical utility in diagnosing and monitoring PH. In our future studies, we aim to functionally verify these differentially expressed miRNAs.

## 5 Conclusion

Of the 136 differentially expressed miRNAs screened in patients with PH, hsa-miR-1304-3p, hsa-miR-490-3p, hsa-miR-11400, and hsa-miR-31-5p were identified as candidate diagnostic biomarkers for PH. GO and KEGG pathway analyses of the genes targeted by these miRNAs revealed that signal transduction and immune-related pathways are notably modulated in PH. Overall, our results provide a new reference and basis for in-depth investigation of the specific mechanisms underlying miRNA-mediated PH regulation.

## Data Availability

The datasets presented in this study can be found in online repositories. The names of the repository/repositories and accession number(s) can be found in the article/Supplementary Material.
